# Editorial: Imaging in Acute Stroke—New Options and State of the Art

**DOI:** 10.3389/fneur.2017.00736

**Published:** 2018-01-11

**Authors:** Anders Fogh Christensen, Hanne Christensen

**Affiliations:** ^1^Department of Radiology, Bispebjerg Hospital, University of Copenhagen, Copenhagen, Denmark; ^2^Department of Neurology, Bispebjerg Hospital, University of Copenhagen, Copenhagen, Denmark

**Keywords:** hyperacute stroke, MRI, CT, sonography, imaging

**Editorial on the Research Topic**

**Imaging in Acute Stroke—New Options and State of the Art**

During the last two decades, the state of art imaging in acute stroke has developed from non-contrast CT performed within 7 days to including hyperacute imaging including both angiographic and perfusion imaging. This includes using both new techniques but also using new ways to combine long existing modalities in daily practice. The increasing focus on the importance of both swift and reliable diagnostics combined with an improved scanner accessibility has fueled this development.

This development in imaging has answered to the needs of the introduction of acute vascular recanalization treatments in ischemic stroke, which has revolutionized the area. I.V. thrombolysis has been increasingly used since the end of the 1990s and is now considered a standard treatment, while mechanical thrombectomy has been accepted as a standard procedure following randomized controlled trials documenting its efficacy within the last 5 years. Further, efficacious treatment options in acute ICH are sought, including thrombostatics to reduce final hematoma volume leading to increased activity in this area also.

The imaging modalities, which are in widespread use in primary stroke imaging—at least in tertiary centers, include CT, MRI, and sonography. These methods are complementary in clinical practice with their different strengths. In the following, we will discuss generally available methods to image brain parenchyma, cerebral, and pre-cerebral vasculature and cerebral perfusion in acute stroke.

## Imaging the Brain Parenchyma

Time is of the essence in the diagnosis and treatment of acute ischemic stroke (1). It has been shown that by using a CT-based set-up for IV thrombolysis, a door to needle time around 20 min is achievable based on admitting patients directly to hospital units providing imaging facilities including radiology department, emergency rooms, or trauma centers (2).

CT has a high sensitivity in detecting blood and thereby identifying a bleeding in the brain parenchyma, which is the major contraindication in IV thrombolysis as well as identifying, e.g., neoplasms. Consequently, IV thrombolysis can be initiated safely based on a non-contrast-CT of the brain as only brain imaging (3).

It has been reported that dual-energy CT (DECT-CT) should improve detection of underlying causes for ICH as well as the differentiation between blood and leaked iodine contrast after endovascular procedures compared to standard non-contrast CT.

Trans cranial Doppler (TCD) has little use in visualizing brain parenchyma but can be used to measure the size of an intracerebral hemorrhage and thus visualize an eventual expansion in size (4); however, the sensitivity for, e.g., hemorrhage in proximity to the scull base does not allow for this modality as only imaging before revascularisation treatment.

MRI is superior to CT in showing acute ischemic changes in the brain parenchyma (5). Diffusion-weighted imaging shows the ischemic lesion in 80–90% of cases in acute stroke but, in the remaining patients, there will not be DWI-positivity, i.e., the DWI-negative stroke (6). Consequently, a reliable diagnosis of stroke cannot be made based on only MRI conformation if ischemia, clinical diagnosis is still needed.

The ability to detect ischemic lesions is, however, also valuable in transient ischemic attack (TIA). This diagnosis is in Europe based on clinical definitions, but severity scores are used in combination with DWI-positivity in the identification of high-risk patients who have a substantial risk of a subsequently ischemic stroke (7). An unexplained sevenfold variation exists in the frequency of DWI lesions between TIA cohorts, and a DWI-negative scan is very far from ruling out a true ischemic event in these patients (8). MRI is also superior in identifying the underlying cause of stroke based on examination of the brain parenchyma: the pattern of DWI lesions helps to differentiate between large vessel disease and cardiac emboli where, in the latter, several vascular territories are often affected. Microvascular changes in small vessel disease can be diagnosed and quantified, not only forming the basis of a diagnosis of small vessel disease but also in differencing between probable cerebral amyloid angiopathy and deep perforator angiopathy. MRI allows for identifying location and number of micro bleeds, lacunar infarcts, unspecific vascular gliosis, and enhanced perivascular space.

Substantial leukoariosis predict a higher risk of both symptomatic and asymptomatic hemorrhage after IV thrombolysis treatment and independent of this was evaluated by MRI or CT (9). The presence of cerebral micro-bleeds before IV thrombolysis treatment predicts and increased risk of new micro-bleeds after treatment. Further, patients with new micro-bleeds were more likely to develop symptomatic remote hemorrhage, but no increase in rate of hemorrhagic transformation was observed, consequently, this is of minor clinical importance (10).

Using MRI in the work-up of hyperacute stroke increases the door to needle time with about 10 min even in a well-organized setting. This is due to MRI safety issues as well as longer scan-time compared to CT (11). A protocol used for evaluating acute ischemia must include T2 flair and a hemo-sequence in order to exclude intracerebral bleeding. MRI is not possible in a substantial number of acute stroke patients due to either safety issues, being unable to cooperate, or in need of close monitoring. The number of patients falling into this category has been reported as high as 20–40% (12) MRI is performed without applying any radiation to the patient in comparison with CT, which makes it safer to use regarding younger patients.

## Imaging the Arterial Vessels of the Head and Neck

By adding CT-angiography (CTA) to an nc-CT, the vessels from the aortic arch to the vertex can be visualized with a resolution of 0.5 mm iso voxel corresponding to a vessel size of 1 mm. This reveals vessel occlusion down to vessel sized too small for mechanical thrombectomy, thereby allowing for precise identification of patients for this procedure. CTA is also reliable in evaluation for underlying pathologies such as dissection and arteriosclerotic disease (13, 14).

CT-angiography in combination with cerebral post-contrast CT is not only a strong tool in identification of neoplasms but also other underlying causes in patients with intracerebral hemorrhage, including some vascular malformations (15). In addition, the presence of arterial and/or venous extravasation of contrast, the so-called spot-sign, is a strong predictor for hematoma expansion with resulting poorer outcome (16). This may be of use in selecting patients for experimental treatment with pro-thrombotic drugs in order to reduce hematoma expansion (16).

Dual-energy CT improves the diagnostic accuracy of CTA in areas close to bone (17), which is clinically highly relevant in patients with posterior circulation stroke.

Sonography can be performed in the stroke unit and, since it involves no radiation exposure, patient safety issues will not limit the number of examinations. It is possible to evaluate both arterial vessels of the neck as well as intracranial vessels—the latter only as a flow examination whereas the vessel wall of the arteries of the neck can be examined with a resolution of 0.2 mm. This allows the detection of occlusions and stenosis as by CTA but with a greater possibility of a non-conclusive examination based on the possible lack of a temporal sonar window or other bone disturbances of the sonar signal. TCD allows for documentation of reperfusion after IV thrombolysis treatment (18) as well as for quantification of collateral status in middle cerebral artery occlusion by measuring the flow in the anterior cerebral arteries (19). This allows for close observation of this critical patient group.

Sonography has the ability to verify vessel wall changes in cervical arteries including dissection, plaque characterization, and detection of unstable thrombi; however, the clinical implications of especially plaque characterization remain uncertain. Likewise, it is standard practice to use Duplex in the preoperative evaluation of carotid stenosis for thrombentaretectomy using flow measurements to quantify the degree of a stenosis even if CTA has documented a reduction in vessel lumen (20). By using ultrasound contrast media, it is possible to diagnose the presence of persistent foramen ovale or a pulmonary fistula if contrast media is detected in the intracerebral circulation after Valsalva (21).

Further, continuous sonographic input on the acute thrombosis increases the thrombolytic effects of rTPA (22).

MR angiography may be performed without contrast as Time of Flight sequences, i.e., arterial TOF, which is more time consuming compared to CT angiography as the scan time is several minutes just to visualize the intracranial arteries and longer if the cervical arteries are also included. The longer the scan time the higher the risk of impairing movement artifacts, which are more prevalent in MRi in an acute setting in comparison to planned examinations (Havsteen et al.).

Contrast-enhanced arterial TOF-sequences improve detection of pathology in the vessel lumen in comparison to non-contrast-enhanced TOF sequences. Contrast-enhanced TOF can be augmented to a resolution down to 0.5 mm isovoxel—the same level as CTA—when using 3-T MRi, but at the expense of prolonged scan time.

Comparative studies show no difference in the sensitivity for detection small aneurisms in comparison to CTA, but the specificity seems to be lower (23). High resolution MRi (HR-MRi) can characterize arteriosclerotic plaques often using contrast-enhanced series, which is reported to give prognostic information about the risk of subsequent vascular events. HR-MRi can also verify dissection changes in small intracranial arteries and visualize reperfusion of such vessels. Vessel wall imaging may be improved by using higher field strength, i.e., 7T MRI (24).

CT-perfusion can be added to the imaging protocol at the price of 5 min more scan-time and an extra 3–5 ms radiation dose. Whole-brain CT-perfusion, i.e., 16 cm coverage has been introduced and has the ability to show areas with low perfusion anywhere in the brain and not restricted to a 4–8 cm slab. This can be useful to both quantify the area with perfusion below which brain parenchyma is lost, i.e., infarct core as well as surrounding salvageable areas with less degree of decreased perfusion, i.e., penumbra. Recently, results from the DAWN study (25) were presented at the ESOC 2017 conference indicating that CT-perfusion—or MR-DWI—can be used to select patients for thrombectomy up to 24 h after ictus, increasing the clinical interest in CTP. The DAWN study was set up to address the issue of wake up and late presenting strokes and neurointervention based on CT-perfusion or DWI-FLAIR mismatch: imaging markers of salvageable tissue. In order to differentiate between ischemic stroke and stroke mimics, perfusion CT may also be helpful and thus compensate for the low visibility of acute ischemia in the brain parenchyma on non-contrast CT alone (26).

An additional feature of whole brain perfusion is the possibility to extract a 4-D angiogram from the perfusion image data without having to perform an angiography; these images show the filling of the intracranial vessels from the early arterial to the late venous phase, which adds a dynamic perspective in the evaluation of acute ischemia improving the visualization of collateral filling (27). The lack of visualization of the vessels of the neck using this method alone can hinder the diagnosis of the underlying cause of stroke.

MRI perfusion is able to show the same findings as CT-perfusion and when it is based on the use of contrast MRI perfusion has considerable advantages especially in obtaining absolute measures of perfusion. It is also possible to apply perfusion sequences without the use of contrast, i.e., arterial spin labeling techniques ASL. ASL has proven as reliable as contrast-enhanced perfusion techniques in detecting areas with reduced perfusion in acute brain ischemia (28) and with the more extensive use of 3T MRi technology, the resolution has improved. It is reported that adding ASL to a DWI-based TIA protocol increases correct detection of ischemic lesions by 5%. This is primarily the case when the DWI lesion is very small and, therefore, doubtful but with a simultaneous area of hypo perfusion surrounding it clarifying the ischemic diagnosis (29).

Perfusion MRI, in combination with DWI, may also guide the decision of mechanical thrombectomy in large vessel occlusion with unknown time of onset based on the mismatch of the infarct core and the surrounding penumbra on CT perfusion (29), but is more time-consuming than the approach used in the DAWN study.

## Conclusion

Combining several image techniques offers several opportunities and advantages. Modern ultrasound apparatus thus offers real-time image fusion so that sonography of the intracranial vessels can be performed using an already performed CTA as anatomical guide. The CTA images can be viewed simultaneously with the live images from TCD and thus assist in the evaluation of intracranial stenosis. Likewise, it is possible in a modern PACS system to co-evaluate all three modalities and thus extract the needed information. This can combine information from a MRI perfusion investigation with CTA visualization of the vessel and Doppler measurements in order to assess the impact of the changes. The information of flow direction and speed form sonography in combination with perfusion measurements and images of the vessel wall can help to explain the extent and location of ischemic lesions and help to decide the treatment options. In acute stroke, the access to all these modalities help to ensure the correct action needed; however, this requires a neuroradiologist on call in collaboration with specialized stroke neurologists. Often in our experience from a tertiary centre, a good strategy is to combine a CTA with an MRI of the brain (DWI, FLAIR, GE/SWI, ASL) to diagnose the cause of stroke and to rule out stroke mimics.

## Author Contributions

This editorial was written with equal participation from the authors.

## Conflict of Interest Statement

The authors declare that the research was conducted in the absence of any commercial or financial relationships that could be construed as a potential conflict of interest.
